# Methylphenidate Efficacy: Immediate versus Extended Release at Short Term in Mexican Children with ADHD Assessed by Conners Scale and EEG

**DOI:** 10.1155/2015/207801

**Published:** 2015-03-08

**Authors:** Alfredo Durand-Rivera, Efren Alatorre-Miguel, Elizabeth Zambrano-Sánchez, Celia Reyes-Legorreta

**Affiliations:** ^1^Laboratorio de Neuroprotección, Servicio de Neurorrehabilitación, Instituto Nacional de Rehabilitación, 14389 México, DF, Mexico; ^2^Centro de Atención Médica y Educación Especial, 14370 México, DF, Mexico; ^3^Laboratorio de Neurofisiología Cognitiva, Instituto Nacional de Rehabilitación, 14389 México, DF, Mexico

## Abstract

Attention deficit hyperactivity disorder (ADHD) affects 5-6% of school aged children worldwide. Pharmacological therapy is considered the first-line treatment and methylphenidate (MPH) is considered the first-choice medication. There are two formulations: immediate release (IR) MPH and long-acting (or extended release) formulation (MPH-ER). In this work, we measure the efficacy of treatment for both presentations in one month with Conners' scales and electroencephalography (EEG). *Results*. for IR group, in parents and teachers Conners test, all items showed significant differences, towards improvement, except for teachers in perfectionism and emotional instability. For ER group in parent's Conners test, the items in which there were no significant differences are psychosomatic and emotional instability. For teachers, there were no significant differences in: hyperactivity and perfectionism. Comparing the Conners questionnaires (parents versus teachers) we find significant differences before and after treatment in hyperactivity, perfectionism, psychosomatics, DSM-IV hyperactive-impulsive, and DSM-IV total. In the EEG the Wilcoxon test showed a significant difference (*P* < 0.0001). As we can see, both presentations are suitable for managing the ADHD and have the same effect on the symptomatology and in the EEG.

## 1. Introduction

Attention deficit hyperactivity disorder (ADHD) is the most commonly diagnosed neurobehavioral disorder in childhood [1, 2], characterized by inattention and/or impulsivity/hyperactivity and emotional instability. It is one of the most prevalent childhood psychiatric disorders, affecting 5-6% of school aged children worldwide [1, 3]. Although there are differences between United States and European diagnostic criteria [4], according to both the Diagnostic and Statistical Manual of the American Psychiatric Association (DSM-IV-TR) and the International Classification of Diseases (tenth edition, ICD-10), ADHD (or hyperkinetic disorder (HKD) according to ICD-10) is characterized by inappropriate levels of inattention, hyperactivity, and impulsivity, which constitute the so-called ADHD core symptoms. These are often accompanied by comorbid symptoms, such as aggressive behaviour, depressive mood, anxiety, and tics, by learning difficulties [5], and by impairment of social functioning [1, 6, 7]. Symptoms impact on their academic achievements and social interaction at school; home life can also be very difficult, as children with ADHD are constantly active and demanding, which tends to cause conflicts with their families. The frequency of referral is higher for boys than for girls (about 2 : 1), and girls are generally older at the time of referral. This is probably because girls are more likely to have the predominantly inattentive subtype of ADHD [8, 9], which, because the symptoms are less striking, is often identified only when poor academic performance is noticed. Symptoms also vary with age and may be less obvious, but equally impairing, in older adolescents. Notable risk factors for developing ADHD include family history of ADHD [8, 10], preterm birth, and smoking or drinking during pregnancy [8, 11].

Pharmacological therapy is considered the first-line treatment for patients with severe ADHD and severe impairment [8, 9]. For decades, ADHD has been treated with stimulant medications, which in most cases produce a rapid and dramatic improvement in ADHD symptoms and in the behavior of affected children [2, 4]. The methylphenidate (MPH) is the first-choice medication for patients without comorbidities, for those with comorbid conduct disorder, and when tics, Tourette's syndrome, anxiety disorder, and stimulant misuse or risk of stimulant misuse are present; however, MPH should be used with caution in such patients. Although the mechanism of action is not completely understood, it is believed to inhibit the reuptake of dopamine and noradrenaline into the presynaptic neuron, increasing their concentration in the extraneuronal space and therefore enhancing neurotransmission [2, 12, 13]. MPH is mainly metabolized by deesterification into ritalinic acid, which is pharmacologically inactive; this results in a short half-life of 2.0–3.0 h and a short duration of action. The maximum plasma concentration (*C*_max⁡_) of MPH and consequently its maximum effect are reached 1.5–2.0 h after dosing [14]. Conventional, immediate release (IR) MPH formulations have been used since 1955 for the treatment of ADHD. Due to the short duration of action, MPH-IR needs to be administered repeatedly during the day to maintain effectiveness, 2-3 daily doses being required for most children [15]. Multiple dosing can be problematic, as it can cause adherence issues and complications related to privacy, stigmatization by classmates, potential abuse, and accountability of the school administration [2]. To overcome these problems, long-acting formulations of MPH have been developed [2, 16, 17]. These MPH-ER formulations provide a rapid onset of therapeutic effect, while having a sufficient duration to eliminate the need for additional doses.

The aim of designing a system controlled release of a drug is to obtain a formulation that releases the active substance at a rate necessary to achieve and maintain a constant concentration in the blood. Such concentration will be or should be similar to that obtained by continuous intravenous infusion wherein the drug is administered to the patient at a constant rate equal to the rate of elimination. This means that the release rate should be independent of the amount of drug remaining in the dosage form and must be constant over given period time [18]. To methylphenidate there are specifications to released amount of the active substance in function of time, since there is a monograph in USP 36 page 1406 about “Methylphenidate Tablets, Extended Release” specifications that are fulfilled by Ritalin LA as well as by Concerta.

For the MPH-ER we are testing Tradea and the formulation is developed from a polymer matrix system where the polymer forming the matrix is hydroxypropyl cellulose (HPC) which is especially used for the controlled release of drugs in hydrophilic matrix systems (inflatable). Compressing a mixture of a relatively soluble drug with the polymer results in a matrix that, in contact with water, hydrates and distends, resulting in a gel through which the drug diffuses. The release of active substance from such systems is due to the contribution of two simultaneous mechanisms: erosion of the outermost layers (and lower gel consistency), dissolution of the active ingredient in the environment and its diffusion through the gel that acts as a barrier. The release rate will depend on the consistency of the polymer gel and the aqueous solubility of the drug, so that when the gel is very weak or the solubility of the drug is very low, the influence of the diffusion to be reduced and the release depends on the erosion rate of the matrix. However, when the drug solubility is moderate or high, the release process can be distinguished in three stages: an initial stage in which this drug is dissolved in the matrix surface and gelation of the polymer begins; a stationary phase during which the water inlet in the matrix produces an expansion of the gel, in which drug release is controlled by diffusion through the gel layer; and finally an exhaustion phase that begins after gelation of the polymer matrix and wherein drug concentration is lower than its solubility coefficient [19]. So the release mechanism of this product and its dissolution profile are not similar to the Ritalin SR, Ritalin LA, or Concerta but meet the specifications of the released amount of active ingredient versus time function, published in USP Monograph 36, page 1406, on “Methylphenidate Tablets, Extended Release” specifications that are fulfilled by Ritalin LA and Concerta.

In this work, we measure the efficacy of treatment for both presentations (immediate release and extended release) at short term (one month) with Conners' scales and electroencephalography.

## 2. Subjects and Method

### 2.1. Instruments

Inclusion criteria were boys and girls, 6 to 18 years, who met the diagnosis of ADHD according to DSM-IV criteria, being drug-treatment-free at the time of the first consultation, no already known diagnosis of a psychotic disorder, severe affective disorder or organic disorder altering their behavior or condition, and having parents who accept the child's income research protocol prior to signing the informed consent.

Elimination criteria were undesirable effects of methylphenidate which are not tolerated by the patient, diagnosis of any comorbidity that warrants the use of any other drug different than methylphenidate, parents or guardians who chose not to continue in the study, or failure to keep an appointment tracking.

After our research project was approved by the Bioethics Clinical Research Committee S. C. in Mexico City and the tutor's or parent's informed consent signature was obtained, the subjects underwent the following tests.

*Subjects.* A total of 38 subjects with probable diagnosis of ADHD has the following applied.

- Before entering the protocol an electroencephalographic study was performed to rule out another condition that was causing the problem (e.g., epileptic focus, absence seizures).
- At the first visit, health history, including vital signs, height, and weight, demographic data (date of birth, gender, and origin), neurological history (fetal distress, severe head injury, and meningitis, including treatment with other drugs since ADHD diagnosis) were checked:
  - in the subsequent three visits (one each week) neurological assessment was performed, including vital signs, weight, and height, questioning any adverse effect or concurrent treatment and verifying the compliance with treatment.
- Conners' scale for parents and teachers was applied, before starting drug treatment and one month after. The parent Conners scale contains 96 questions grouped into 8 factors:
  - alterations of conduct,
  - fear,
  - anxiety,
  - restless-impulsivity,
  - immaturity-learning problems,
  - psychosomatic problems,
  - obsession,
  - antisocial behaviors,
  - hyperactivity.
- The Conners scale for teachers is much shorter and consists of 39 questions grouped into 6 factors:
  - hyperactivity,
  - behavior problems,
  - emotional instability,
  - anxiety-passivity,
  - antisocial behavior,
  - Difficulties in sleep.
- Each question describes a characteristic behavior of these children that parents or teachers should assess, according to the intensity with which it arises (none = 0, little = 1, quite = 2, and a lot = 3).
- Electroencephalographic records: an electroencephalograph brand “Aconic,” model BioPC of 32 channels, was used with a speed of 3 cm/s. All recorders were conducted in wakefulness with eyes closed. At first, whether the EEG was normal or abnormal was indicated. That was considered normal EEG study when the rhythms observed symmetry, synchrony, and the topography, according to the age, with no abnormal graphoelements. The EEG data of immaturity considered were those that had a base rhythm inconsistent with the patient's age, with poor structuring backbeat, or that do not comprise an age appropriate pace. Other abnormalities were categorized in encephalopathy grade I, grade II, and grade III, according to the degree of abnormality (American Clinical Neurophysiology Society, Guideline 7: Guidelines for Writing EEG Reports: “If the record is considered abnormal, it is desirable to grade the abnormality in order to facilitate comparison between successive records for the person who receives the report. Since this part of the report is largely subjective, the grading will vary from laboratory to laboratory, but the different grades should be properly defined”).
- Randomization: as patients entered the protocol, they were assigned immediate release (IR) or extended release (ER) treatment according to systems 1-2 consecutively.
  - The dose was methylphenidate (Tradea) 1 mg/kg/day.
- We performed Student's *t* tests for related samples Conners scales for both parents and teachers; likewise, Student's *t* test was performed for independent samples to compare results of parents versus the teachers, before and after treatment and comparing IR versus ER groups, before and after treatment. For the EEG, a descriptive analysis was performed first and after the Wilcoxon test.

## 3. Results

Of the 38 subjects, 28 completed the protocol, 2 did not meet the criteria for the diagnosis of ADHD, for one, the father decided to withdraw informed consent, 6 were withdrawn for not attending follow-up appointments, and one had probable adverse effect of the drug.

Of the 28 patients who completed the protocol, 21 patients were male (75%) and 7 females (25%); the average age was 9 years 2 months, height 1.37 m, and weight 35.750 kg.

Patients assigned to the IR group were 15, 12 males (80%) and 3 women (20%); the averages were as follows: age, 9 years 2 months, height, 1.35 m, and weight, 34.780 kg. Patients assigned to the ER group were 13, 9 males (69.2%) and 4 females (30.8%) and the averages were age of 9 years 3 months, height of 1.39 m, and weight of 36.869 kg.

**Figure 1:**
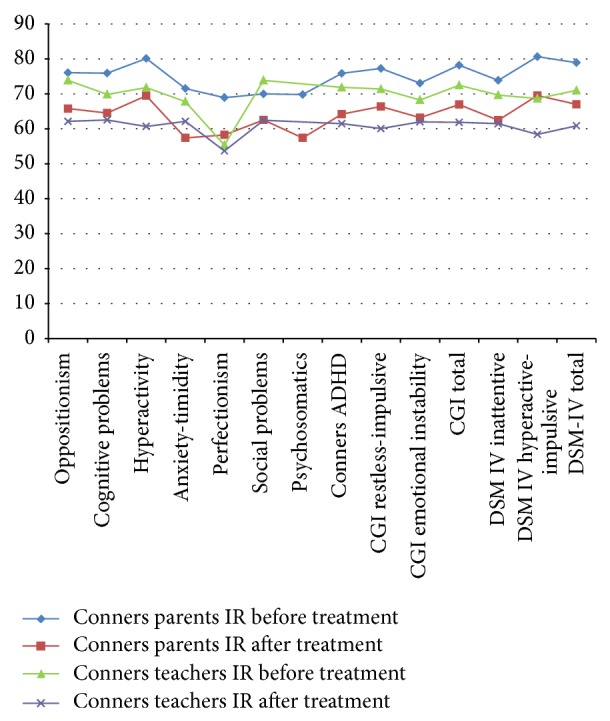


For IR group patients, in relation to parent's Conners test, in all items there were significant differences toward improvement (Table 1) (Figure 1). For teachers the only items where there were no significant differences are CGI perfectionism and emotional instability (Table 1) (Figure 1).

**Figure 2:**
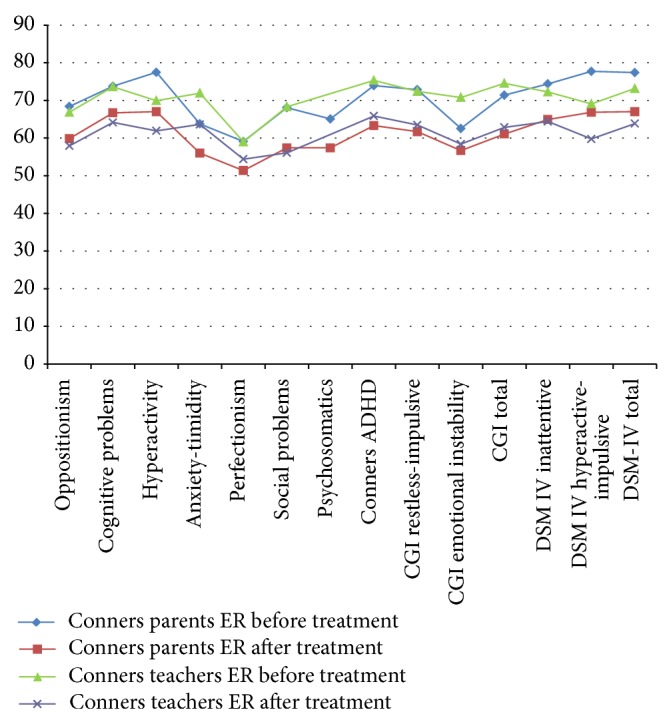


For ER group patients, with respect to parent's Conners test, the items where there was no significant difference are psychosomatics and emotional instability CGI (Table 2) (Figure 2). For teachers, there were no significant differences in hyperactivity and perfectionism (Table 2) (Figure 2).

In comparing the Conners questionnaires, parents versus teachers, we find significant differences before treatment in hyperactivity, perfectionism, psychosomatics, DSM-IV hyperactive-impulsive, and DSM-IV total. After treatment differences were found in the same items except perfectionism, which disappears (Table 3).

When comparing the IR versus ER groups in parent's Conners scales, significant differences were found before treatment in CGI perfectionism and emotional instability, which cease to exist after treatment (Table 4). For teachers no significant differences existed in either the first or the second time.

In the case of the EEG first it was a descriptive analysis and then the Wilcoxon test, which showed a very significant difference (*P* < 0.0001) (Table 5).

The adverse effect profile of the formulation of methylphenidate (Tradea) observed in the protocol is similar to that described in the literature (see Table 6), such effects being mild, well known, transient, and related to the drug.

## 4. Discussion

The MTF is a psychostimulant which preferential action on dopaminergic pathway gets better attention and inhibitory control of the impulse, implementing executive function, academic performance and behavior; is indicated as part of a comprehensive treatment program for ADHD in children over 6 years and adolescents when remedial measures by themselves are inadequate. 65 to 75% of ADHD patients respond to stimulant substances compared with 4 to 30% response rate to placebo [20, 21]. The treatment should be done under medical supervision of a specialist in behavioral disorders in childhood. The diagnosis has to be made according to DSM-IV criteria or the guidelines in ICD-10. In this regard the trend of ICD-10 to generate false negatives for pure inattentive and DSM-IV to give false positives in mild cases is worth recalling [22].

Methylphenidate is rapidly absorbed orally, starting to act 20–30 minutes after ingestion, and is eliminated relatively quickly, so that the effect is only maintained for 3 or 4 hours. Specialists believe that in those children the drug effect lasts more than four hours because of psychological factors and not the drug itself. Methylphenidate, unlike other drugs, has no major side effects; certain sleep problems and decreased appetite have only been recorded in some studies [20, 22]. In our case only one patient dropped out due to probable adverse effect that was nervous and trembling, but it was found that no direct effect of the drug existed; 2 patients showed a lack of appetite and mild drowsiness during the first week; but this did not cause the withdrawal of the drug and the symptoms disappeared later.

MPH's mechanism of action is not completely understood but is known to block receiving norepinephrine and dopamine in the presynaptic neuron, thereby increasing the levels of these substances in the extraneuronal space. Orally, the MPH is rapidly absorbed in the gastrointestinal tract and its maximum blood concentration is obtained between 1 and 3 hours after ingestion. The MPH has a short half-life (2-3 hours) and also short effect (3-4 hours). Therefore, in an immediate release formulation, it requires two or three daily administrations of the drug, which causes fluctuations in plasma concentrations (pulsatile pattern). This pattern allows precise titration of dosage, is effective in controlling ADHD symptoms when the patient needs it most, allowing very flexible settings for each patient dose, and has an optimal cost-effectiveness. The doses commonly used are ranging from 0.3 mg to 1.5 mg/kg/day [21]. On the other hand the development of delayed or sustained release allows maintenance of blood levels of MPH, without the variations of the fast formulation, and the convenience of the single daily dose. The MPH is released gradually during the following hours, but has the problem that you cannot breach or masticate the tablets [21].

In our work we wanted to see if there were differences between the uses of MPH immediate release and the MPH extended release in a short period of time (1 month), measuring this impact with the Conners scale that measures household behaviors through the questionnaire for parents and school behaviors through the questionnaire for teachers.

Seeing that virtually there is no difference between submission IR and ER and as we can see in Table 1 and Figure 1 with regard to the parents in the presentation IR improvement was observed in all the items and with regard to the presentation LP, the only items where changes toward improvement were not statistically significant were: the psychosomatic and emotional instability items where both behaviors are often an addition to the pathology of ADHD, and no part of it pathology. However, if you review the data when comparing groups IR versus ER before initiating treatment, there were significant differences with regard to emotional instability (Table 4) and those differences are lost after the treatment, which means that the improvement in both groups was similar.

With regard to teachers, there were no significant differences between the two presentations (IR versus ER), meaning that both have the same effect regarding school behavior (Table 4). For IR presentation, for teachers, it is observed that there were no significant differences in perfectionism and emotional instability, whereas for the ER presentation, differences were found in the areas of hyperactivity and perfectionism (Tables 1 and 2, Figures 1 and 2), so we can see with this that, for a short time, teachers do not have the opportunity to verify such behavior because normally they are based on the overall performance of the group with respect to the child with ADHD and these items are difficult to evaluate for them.

When comparing the Conners questionnaires, parents versus teachers, we find significant differences in pretreatment hyperactivity, perfectionism, psychosomatics, DSM-IV hyperactive-impulsive, and DSM-IV total and these differences remain after treatment with the exception of perfectionism which disappears (Table 3); likewise, if we look at the averages before and after treatment, we realize that all items were reduced averages which means that children improved their behavior; however apparently parents and teachers observe these behaviors in different way in the children; that is to say about the results, parents are more severe in judging the behavior of children (Table 3), perhaps for not having the perspective of the group.

On the other hand, the electroencephalogram (EEG) has no value from the point of view of ADHD diagnosis, but a scan that has no risks and gives us valuable information on how the brain electrical activity worked so as not to forget that this is the most common neurodevelopmental disorder, and also it has described paroxysmal EEG abnormalities in children with ADHD, in some series, reaching 15–20% of cases [21, 23]. Moreover, at the slightest suspicion that attention problems may be related to episodes of “absences,” it must make an EEG study. In any case, should not systematically ignored the EEG, since it can show relevant changes that surprise us in patients who have never had crisis and at least have to be careful to inform parents and reconsider the advisability of giving a psychostimulant when frequent epileptic paroxysms occur in a child with ADHD, being then most prudent, think of a drug that has no psychostimulating effects. We must not forget that children with epilepsy have disorders of attention mechanisms much more frequently [24, 25] and, moreover, these disorders are those that affect learning problems and complicate the lives of these children and their parents. Recall that epilepsy includes not only manifestations of seizures, and therefore in epileptic children is very convenient to make a complete neuropsychological evaluation that includes the study of attentional mechanisms. In any case, a well-controlled epileptic patient, with no active epilepsy, can take psychostimulant drugs, if this positively affects the clinical outcome of ADHD symptoms [26].

In our case no child showed electroencephalographic activity of epileptiform type; however, only 7.14% of the studies conducted in our sample do not have altered electroencephalographic and the rest (92.86%) had some type of bioelectrical alteration that was not of epileptic type. Cornelio-Nieto et al. report that the EEG changes that occur in patients with ADHD are nonspecific and are not necessarily epileptogenic foci, as many patients with these findings will never present with seizures and, probably, the observed EEG changes in children with ADHD only reflects in these specific cases disturbance of cerebral bioelectric operation which is not observed in the majority of children with ADHD [27]. We agree with them regarding the EEG changes only reflect alterations in brain bioelectrical operation; however, in our case most children showed these changes, which means that most children with ADHD may have EEG alterations that are not being taken into account by not being clearly epileptic. However, despite the short time of treatment, 92.86% of our children who had some type of electroencephalographic alteration regardless whether treatment was with IR or ER showed a favorable change in the activity, which implies that the drug is being used to assist in organizing the child's brain electrical activity, which is reflected in the behavior of the same child (Table 5, Figures 1 and 2). This is very important because the encephalographic record can serve to objectively monitor, with questionnaires and neuropsychological tests, the efficacy of psychostimulant drug, in the case of any alteration electroencephalographic not being epileptogenic type, which in our case was very common.

As we can see from the results, both the IR-MPH and the ER-MPH are suitable for handling the ADHD; properly used, both presentations have the same effect on the symptomatology both at school and at home and in our case it shown in the EEG too.

The advantages of IR-MPH are its flexibility and management of doses that can be given during the day. The advantages of long-acting ER-MPH reside in a lower risk of abuse and, above all, a lower number of shots, resulting in better compliance, less need of other persons like in the school and for doses, and less stigmatization. Therefore, each drug has its niche indication.

This allows giving each patient the necessary medication on their schedule and needs. Undoubtedly, certain children will benefit from the exclusive making of a long-acting drug; others will only require immediate action doses and others a combination of both. This is even truer if we say that a particular child may require a type of medication on weekend and another on Monday to Friday, as the schedules and activities are completely different.

## Figures and Tables

**Table 1:** 

	Parents IR					Teachers IR				
	Conners (before)		Conners (after)			Conners (before)		Conners (after)		
	Mean	Standard error	Mean	Standard error	*P*	Mean	Standard error	Mean	Standard error	*P*
Oppositionism	76.07	2.959	65.80	2.717	**0.000**	73.87	3.916	62.13	3.386	**0.005**
Cognitive problems	75.93	1.855	64.53	1.961	**0.000**	69.87	2.388	62.53	2.596	**0.001**
Hyperactivity	80.13	2.410	69.47	2.546	**0.001**	71.80	3.048	60.67	2.939	**0.001**
Anxiety, timidity	71.53	3.319	57.40	2.507	**0.000**	67.80	2.701	62.13	2.167	**0.029**
Perfectionism	68.93	2.652	58.27	3.130	**0.000**	55.33	2.772	53.67	2.081	0.382
Social problems	70.00	2.776	62.53	2.744	**0.019**	73.87	3.380	62.47	3.142	**0.002**
Psychosomatics	69.80	4.208	57.40	3.494	**0.004**					
Conners ADHD	75.8667	1.788	64.20	1.547	**0.000**	71.87	2.095	61.47	2.054	**0.001**
CGI restless-impulsive	77.27	2.260	66.40	1.558	**0.001**	71.40	2.408	60.07	1.703	**0.000**
CGI emotional instability	73.07	2.843	63.20	3.060	**0.000**	68.27	4.677	62.00	3.117	0.094
CGI total	78.20	2.232	66.93	1.972	**0.000**	72.47	2.867	61.87	2.056	**0.001**
DSM IV inattentive	73.87	1.968	62.47	1.841	**0.000**	69.67	2.179	61.47	2.404	**0.001**
DSM IV hyperactive-impulsive	80.67	2.472	69.53	2.297	**0.001**	68.67	3.007	58.40	2.806	**0.000**
DSM IV total	78.93	1.909	67.00	1.702	**0.000**	71.00	2.120	60.87	2.184	**0.000**

IR = immediate release.

Significance = *P* < 0.05.

**Table 2:** 

	Parents ER					Teachers ER				
	Conners (before)		Conners (after)			Conners (before)		Conners (after)		
	Mean	Standard error	Mean	Standard error	*P*	Mean	Standard error	Mean	Standard error	*P*
Oppositionism	68.38	3.522	59.85	3.861	**0.005**	66.85	4.883	57.92	3.343	**0.004**
Cognitive problems	73.77	2.827	66.69	2.960	**0.005**	73.62	1.243	64.15	1.750	**0.000**
Hyperactivity	77.46	2.645	67.00	3.258	**0.003**	69.92	4.202	61.92	3.480	0.063
Anxiety, timidity	63.77	4.442	56.00	4.486	**0.001**	71.92	3.348	63.62	3.680	**0.018**
Perfectionism	59.08	4.002	51.38	3.748	**0.001**	59.00	4.103	54.38	3.719	0.207
Social problems	68.08	3.104	57.38	3.253	**0.009**	68.31	3.426	56.08	2.894	**0.002**
Psychosomatics	65.08	4.055	57.38	3.447	0.074					
Conners ADHD	73.92	2.702	63.31	2.466	**0.000**	75.31	2.841	65.85	3.044	**0.025**
CGI restless-impulsive	72.85	3.002	61.69	2.263	**0.001**	72.38	3.025	63.46	3.054	**0.033**
CGI emotional instability	62.54	3.738	56.69	2.502	0.151	70.77	4.147	58.38	3.167	**0.024**
CGI total	71.38	2.949	61.08	2.288	**0.001**	74.54	3.153	62.85	2.729	**0.005**
DSM IV inattentive	74.38	2.742	64.92	3.029	**0.004**	72.23	2.023	64.38	1.920	**0.003**
DSM IV hyperactive-impulsive	77.69	2.744	66.85	3.425	**0.003**	69.08	4.034	59.77	3.523	**0.021**
DSM IV total	77.38	2.490	67.00	3.132	**0.002**	73.15	2.937	63.85	2.606	**0.008**

ER = extended release.

Significance = *P* < 0.05.

**Table 3:** 

Item	Before		Significance (bilateral)	After		Significance (bilateral)
	Parents	Teachers		Parents	Teachers	
Oppositionism	72.50	70.61	0.629	63.04	60.18	0.395
Cognitive problems	74.93	71.61	0.131	65.54	63.29	0.339
Hyperactivity	78.89	70.93	0.012	68.32	61.25	0.022
Anxiety-timidity	67.93	69.71	0.611	56.75	62.82	0.061
Perfectionism	64.36	57.04	**0.038**	55.07	54.00	0.738
Social problems	69.11	71.29	0.495	60.14	59.50	0.834
Psychosomatics	67.61	.00	0.000	57.39	.00	0.000
Conners ADHD	74.96	73.46	0.522	63.79	63.50	0.901
CGI restless-impulsive	75.21	71.86	0.209	64.21	61.64	0.244
CGI emotional instability	68.18	69.43	0.755	60.18	60.32	0.963
CGI total	75.04	73.43	0.572	64.21	62.32	0.410
DSM IV inattentive	74.11	70.86	0.146	63.61	62.82	0.735
DSM IV hyperactive-impulsive	79.29	68.86	**0.001**	68.29	59.04	**0.003**
DSM IV total	78.21	72.00	**0.010**	67.00	62.25	**0.050**

Significance = *P* < 0.05.

**Table 4:** 

Item	Parents IR	Parents ER	Significance	Teachers IR	Teachers ER	Significance
Oppositionism	76.07	68.38	0.104	73.87	66.85	0.267
Cognitive problems	75.93	73.77	0.517	69.87	73.62	0.195
Hyperactivity	80.13	77.46	0.461	71.80	69.92	0.716
Anxiety-timidity	71.53	63.77	0.167	67.80	71.92	0.342
Perfectionism	68.93	59.08	0.045	55.33	59.00	0.455
Social problems	70.00	68.08	0.647	73.87	68.31	0.261
Psychosomatics	69.80	65.08	0.430	.00	.00	0.000
Conners ADHD	75.87	73.92	0.544	71.87	75.31	0.331
CGI restless-impulsive	77.27	72.85	0.243	71.40	72.38	0.799
CGI emotional instability	73.07	62.54	0.031	68.27	70.77	0.696
CGI total	78.20	71.38	0.073	72.47	74.54	0.630
DSM IV inattentive	73.87	74.38	0.877	69.67	72.23	0.401
DSM IV hyperactive-impulsive	80.67	77.69	0.427	68.67	69.08	0.935
DSM IV total	78.93	77.38	0.621	71.00	73.15	0.550

IR = immediate release.

ER = extended release.

Significance = *P* < 0.05.

**Table 5:** 

EEG results		
Diagnosis	Before Tx	One month after Tx
Normal	2	8
Immaturity	1	6
Encephalopathy grade I	9	9
Encephalopathy grade II	12	5
Encephalopathy grade III	4	0

Total	28	28

Tx = treatment.

**Table 6:** Reporting of adverse reactions and effects.

Patient number	Release type (extended/immediate)	Adverse effect
1	ER	Lack of appetite Constipation
2	ER	Lack of appetite Weariness Apathy
8	ER	Lack of appetite Nausea
15	IR	Vomiting
17	IR	Drowsiness
26	ER	Nervousness Tremors
31	ER	Myokymia

(i) ER = extended release.

(ii) IR = immediate release.

The adverse effect profile of the formulation of methylphenidate (Tradea) observed in the protocol is similar to that described in the literature, such effects being mild, well known, transient, and related to the drug.
